# Rapid and recent diversification patterns in Anseriformes birds: Inferred from molecular phylogeny and diversification analyses

**DOI:** 10.1371/journal.pone.0184529

**Published:** 2017-09-11

**Authors:** Zhonglou Sun, Tao Pan, Chaochao Hu, Lu Sun, Hengwu Ding, Hui Wang, Chenling Zhang, Hong Jin, Qing Chang, Xianzhao Kan, Baowei Zhang

**Affiliations:** 1 School of Life Sciences, Anhui Key Laboratory of Eco-engineering and Bio-technique, Anhui University, Hefei, Anhui, China; 2 School of Life Science, Nanjing Normal University, Nanjing, Jiangsu, China; 3 Key Laboratory for Plant Diversity and Biogeography of East Asia, Kunming Institute of Botany, Chinese Academy of Sciences, Kunming, Yunnan, China; 4 College of Life Sciences, Anhui Normal University, Wuhu, Anhui, China; 5 Faculty of Life Science and Chemical Engineering, Jiangsu Second Normal University, Nanjing, Jiangsu, China; 6 Department of Molecular and Cell Biology, University of Leicester, Leicester, United Kingdom; National and Kapodistrian University of Athens, GREECE

## Abstract

The Anseriformes is a well-known and widely distributed bird order, with more than 150 species in the world. This paper aims to revise the classification, determine the phylogenetic relationships and diversification patterns in Anseriformes by exploring the Cyt *b*, ND2, COI genes and the complete mitochondrial genomes (mito-genomes). Molecular phylogeny and genetic distance analyses suggest that the *Dendrocygna* species should be considered as an independent family, Dendrocygnidae, rather than a member of Anatidae. Molecular timescale analyses suggests that the ancestral diversification occurred during the Early Eocene Climatic Optimum (58 ~ 50 Ma). Furthermore, diversification analyses showed that, after a long period of constant diversification, the median initial speciation rate was accelerated three times, and finally increased to approximately 0.3 sp/My. In the present study, both molecular phylogeny and diversification analyses results support that Anseriformes birds underwent rapid and recent diversification in their evolutionary history, especially in modern ducks, which show extreme diversification during the Plio-Pleistocene (~ 5.3 Ma). Therefore, our study support that the Plio-Pleistocene climate fluctuations are likely to have played a significant role in promoting the recent diversification for Anseriformes.

## Introduction

Adaptive radiation, the evolution of ecological and phenotypic diversity within a rapidly multiplying lineage, has fascinated biologists over the past century [1–3]. Identifying the evolutionary diversification of organisms is a crucial step in understanding their evolutionary history, where the final goal is exploring the factors that might potentially affect the diversification [4]. It was known that molecular phylogenies provide robust framework to study the patterns of speciation and diversification in lineages [5–6]. The branching pattern of a phylogenetic tree can be used to detect changes in speciation through time [7]. This information can also be used to detect speciation bursts and identify historical factors underlying the emergence of ecological and phenotypic divergence within a lineage [8]. Up to now, rapid radiation and burst speciation events have been examined in several taxonomic groups, such as fishes [8, 9] and mammalian [4, 10–12]. Notably, an ancient, rapid radiation event was also observed in the evolutionary history of the Galliformes species [13]. Furthermore, hybridization is a common phenomenon when populations invade new environments and potentially elevates rates of response to selection, which plays an important role in species evolutionary processes, such as adaptive radiations [14]. Particularly, birds show relatively high levels of hybridization [15, 16], such as the high incidence of hybridization in Anseriformes birds (e.g. ducks, geese and swans) [17, 18].

Anseriformes is a large and well-known bird group comprising more than 150 species (approximately 43 genera), including ducks, geese, swans, screamers and the magpie goose [19, 20]. In the past few decades, the Anseriformes species have been among the most studied groups of birds, especially regarding phylogenetic relationships [18, 19, 21–26]. However, the classification of the Anseriformes has been much disputed and revised since the recognition of this group [27–30]. Originally, the order Anseriformes has been considered to be composed of the families Anhimidae (screamers) and Anatidae (ducks, geese, swans and the magpie goose) [21, 31–35]. Currently, the Anseriformes has been divided into three families: Anatidae (ducks, geese and swans), Anhimidae (screamers) and Anseranatidae (the magpie geese) [20, 36–41]. However, some researchers suggest that *Dendrocygna* species should be considered as an independent family [42, 43]. Besides, the classification of several genera in Anatidae, such as the *Amazonetta*, *Speculanas*, *Tachyeres* and *Lophonetta*, has been much debated [44–48].

Compared with nuclear DNA, the mitochondrial genome (mito-genome) of animals evolve rapidly [49]. Previous studies proved that the mitochondrial DNA (mtDNA) sequences are a powerful tool for taxonomy, phylogenetic relationships, evolutionary history, etc [10, 28, 50–56]. However, single-gene phylogenies often differ dramatically from studies involving multiple datasets, which suggest that they are often discordance [57]. Therefore, the mito-genome and concatenated genes (mitochondrial gene or nuclear gene) were gradually used to construct reliable phylogeny for determining evolutionary relationships among species or higher taxa [10–13, 57–62]. Currently, the mito-genomes or concatenated mitochondrial genes, such as NADH dehydrogenase subunit 2 (ND2), Cytochrome *b* (Cyt *b*), cytochrome oxidase subunit I (COI) and 12S ribosomal RNA (12S rRNA), etc, were widely used for systematics studies in the bird’s evolution [19, 27, 28, 56, 58, 63]. What’s more, with the development of the sequencing, the use of nuclear genes or whole genomics for systematics studies is increasing [11, 25, 26, 64–66].

In the present study, we set out to examine the phylogenetic relationships and revise the classification in the Anseriformes based on mito-genomes (Dataset 1), two concatenated mitochondrial genes (Cyt *b* and ND2, Dataset 2) and the DNA barcoding gene (COI, Dataset 3). Generally, the diversification pattern of species is not always gradual but can occur in rapid radiations, especially after major environmental changes [67, 68]. In this study, we also want to determine whether Anseriformes birds experienced rapid diversification and explore the potential factors that may have been an influence. Therefore, we performed diversification analyses to further contribute to an understanding of the diversification patterns in Anseriformes.

## Materials and methods

### Ethical statement

In the present study, all samples were collected from individuals that died naturally and were found in field investigations. Our experimental procedures complied with the current laws on animal welfare and research in China, and were specifically approved by the Animal Research Ethics Committee of Anhui University.

### Sample collection and DNA extraction

From 2006 to 2013, muscle samples of nine Anatidae species (*Anas acuta*, *A*. *poecilorhyncha*, *A*. *crecca*, *A*. *clypeata*, *Aythya ferina*, *Aythya fuligula*, *Mergus merganser*, *Tadorna tadorna* and *Aix galericulata*) were collected from the Anqing wetland along the Yangtze River in Anhui Province. Total genomic DNA was extracted using a standard proteinase K / phenol-chloroform protocol, as described by Sambrook & Russell [69]. An EasyPure PCR Purification Kit (TransGene) was used to purify each DNA extraction.

### PCR amplification and sequencing

Sixteen pairs of primers (S1 Table) were designed using Primer Premier 5.0 [70] based on the mito-genomes of *Anas platyrhynchos* (EU009397) [71], *A*. *falcata* (NC_023352) [72], *Anser fabalis* (NC_016922) [27], *Dendrocygna javanica* (NC_012844) [73] and *Anseranas semipalmata* (NC_005933) [74]. The product size of the above primer pairs ranged from 1,137 bp to 1,537 bp (S1 Table). Polymerase chain reaction (PCR) was performed in a 50 μL reaction mixture, containing 100 to 200 ng of genomic DNA, 25 μL 2×Easy*Taq* PCR SuperMix polymerase (TransGen Biotech, containing 1.25U Ex *Taq*, 0.4mM dNTP, 4mM Mg^2+^) and 0.4 μM of each primer. Pure molecular biology grade water was added to reach the appropriate volume. Thermal cycling consisted of a denaturation step at 95°C for 5 min, followed by 30 cycles of denaturation (95°C, 30 s), annealing (50–55°C, depending on primer combinations, 40 s) and extension (72°C, 90 s) and a final extension step of 10 min at 72°C. PCR products were purified using an EasyPure PCR Purification Kit (TransGene), and sequenced using previous primers and the BigDye Terminator v3.0 Ready Reaction Cycle Sequencing Kit (Applied Biosystems) following the manufacturer’s instructions on an ABI PRISM 3730 automated sequencer. Several different methods (e.g. BLAST search and translation test method) had been adopted to exclude potential nuclear mitochondrial pseudogenes [75].

### Sequence analyses

Sequences were assembled by Seqman II (DNAStar, Madison, WI, USA) and examined visually to ensure the accuracy of variable sites identified by the program [76]. Protein-coding genes (PCGs) were identified by comparison with known complete mtDNA sequences of Anseriformes birds using Sequin 11.0. The 22 tRNA genes were identified using the software package tRNA Scan-SE 1.2.1 (http://lowelab.ucsc.edu/tRNAscan-SE/) [77]. The graphical map of the mito-genome was drawn using the online software OrganellarGenomeDRAW (http://ogdraw.mpimp-golm.mpg.de/) [78]. In addition, the DOGMA annotation software was used to check annotated genes [79]. All assembled and annotated mito-genomes have been deposited in GenBank (Accession no. KF312717, KF156760, KF203133, KT345702, KJ710708, KJ722069, KU140667, KU140668 and KJ169568) [80–86]. Other mtDNA sequences used in the analyses were downloaded from the NCBI database (http://www.ncbi.nlm.nih.gov/pubmed/) (S2, S3 and S4 Tables for a full list accession numbers of sequences). Genetic distances based on the COI gene among species were calculated using the Kimura two parameter (K2P) distance model [87] performed in MEGA5 [88] with 10,000 replications.

### Phylogenetic analyses

Phylogenetic trees were reconstructed based on the mito-genomes of 30 Anseriformes species (Dataset 1, S2 Table). In this study, Dataset 1 were estimated under the Maximum Likelihood (ML) and Bayesian Inference (BI) methods, with *Gallus gallus* (NC_001323) used as an out-group, which were performed with RAxML version 8 [89] and MrBayes 3.2.2 software program [90], respectively.

Before phylogenetic tree constructions, sequence alignment was carried out by using Clustal X 1.8 software [91] and examined visually. To obtain the estimated best fit evolution model for each data set, we performed analyses separately as described above using the MrModeltest 1.0b software program [92] in Paup* 4.0b [93], which was based on the AIC criterion. For ML analysis, node support was calculated with a GTRGAMMA model via rapid bootstrapping with ten runs and 1,000 replications to estimate the best topology. For the BI analysis, two independent runs of four Markov Chains Monte Carlo (MCMC) chains (one cold chain and three hot chains) were simultaneously run under the best fit substitution model (GTR + *I* + *G*) for 1,000,000 generations, with sampling conducted every 100 generations until the average standard deviation of split frequencies reached a value less than 0.01. The first 10% of the total trees were discarded as ‘‘burn-in” and the remaining trees were used to calculate 50% majority rule consensus tree and Bayesian posterior probabilities.

### Dating analyses

To estimate the precise divergence times within Anseriformes, we dated the divergence time between Anseriformes and Galliformes with multiple out-groups (*Columba livia*, NC_013978 [94]; *Falco peregrinus*, NC_000878 [95]; *Tinamus guttatu*s, NC_027260 [96] and *Struthio camelus*, NC_002785 [96]). All calibration points used in the dating analyses were derived from Jarvis’ study [66] (S5 Table). Then, we used the divergence time between Anseriformes and Galliformes as the calibration point to estimate the divergence within Anseriformes. We applied a Bayesian MCMC method based on the mito-genomes, which employs a relaxed molecular clock approach, and implemented in BEAST 1.7.4 [97, 98]. A relaxed uncorrelated log normal model of lineage variation and a Yule Process prior to the branching rates based on HKY + *I* + *G* model were assumed, as recommended by MrModel test 1.0b. Four replicates were run for 1,000,000 generations with tree and parameter sampling that occurred every 100 generations for the first 10% of samples that were discarded as burn-in. All parameters were assessed by visual inspection using Tracer v. 1.6 [99]. To further estimate the divergence times within the Anseriformes, we also used concatenated sequences (ND2 and Cyt *b* sequences) for 128 species (Dataset 2, S3 Table), and were based on the above calibration points (S5 Table). Compared with Gonzalez’s study [17], seven species (*Tachyeres brachypterus*, *T*. *leucocephalus*, *Mergus squamatus*, *Dendrocygna arcuata*, *Anhima cornuta*, *Chauna torquata* and *Anseranas semipalmata*) were added. Relaxed uncorrelated log normal models of lineage variation and the Yule Process were set by basing them on HKY + *I* + *G* model as recommended by MrModel test 1.0b.

### Tempo and rate shifts of diversification analyses

To visualize the temporal accumulation of lineages, a log-transformed lineage-through-time (LTT) plot [100] was performed based on 1,000 trees randomly selected from the BEAST analysis by using APE package (version 4.1) [101] in R language [102]. In this study, the LTT plots were based on the molecular phylogeny of 128 species in Anseriformes (excluding out-groups).

BAMM is a Bayesian approach that uses reversible jump Markov chain Monte Carlo (rjMCMC) sampling to explore shifts in macro-evolutionary regimes assuming they occur across the branches of a phylogeny under a compound Poisson process, and explicitly accommodates diversification rate variation through time and among lineages [103, 104]. Therefore, we used the program BAMM to estimate rates of speciation, conduct rate-through-time analysis of these rates, and identify and visualize shifts in species rates across the molecular phylogeny.

BAMM accounts for non-random and incomplete taxon sampling in the phylogenetic trees by allowing all non-sampled species to be associated with a particular tip or more inclusive clade [103, 104]. Therefore, we assumed that our sampling included 80% of extant Anseriformes species diversity (4 families, 39 genera and 128 species in total; S3 Table). Furthermore, priors for BAMM were generated using setBAMMpriors function, implemented in R package BAMMtools v 2.1.6 [103], by providing the Maximum Clade-Credibility (MCC) tree from BEAST and total species numbers across the order (approximately 160) [41]. Four independent MCMC chains of 20,000,000 generations, sampling event data every 20,000 steps, were run in BAMM and convergence was assessed by computing the effective sample sizes of log likelihoods, as well as the number of shift events present in each sample using the R package coda v. 0.16–1 [105]. After removing 10% of trees as burn-in, we analyzed the BAMM output using BAMMtools and computed the 95% credible rate shift configurations using the summary of posterior distribution of the number of shifts.

## Results

### Mito-genome organization

In this study, nine mito-genomes were sequenced and annotated, which contain 13 PCGs (including ATP6, ATP8, COI, COII, COIII, ND1, ND2, ND3, ND4, ND4L, ND5, ND6 and Cyt *b*), two rRNAs (12S rRNA and 16S rRNA), 22 tRNAs and a control region (CR), respectively. The total length of these mito-genomes ranged from 16,599 bp to 16,630 bp. The heavy DNA strand (H-strand) carried most of the genes (12 PCGs, two rRNAs and 14 tRNAs), while ND6 and eight tRNAs were located on the L-strand (S1 Fig). The arrangement of the whole mito-genome of these species were identical to known typical vertebrate patterns [106]. The total length of the 13 PCGs was 11,411 bp, which represented approximately 68.7% of the entire mito-genome in Anatidae. The overall base composition is also similar to other Anseriformes species. For example, A + T content (50.6–51.8%) (S6 Table) is higher than C + G content, which reflects the strong AT bias [107]. What’s more, the relative abundance of nucleotides is C > A > T > G, which showed that Guanine (G) is the rarest nucleotide (S6 Table), similar to other vertebrate animals [48, 49, 107].

### Genetic distance

To understand the sequence divergence within the Anseriformes, we calculated genetic distances among different groups based on COI gene for 54 species. In this study, the K2P distances among four families ranged from 0.158 to 0.208, while the average genetic distances were 0.117 ± 0.015 (Table 1, Dataset 3). In addition, the K2P distances among genera within Anatidae ranged from 0.037 to 0.107, while the average genetic distances were 0.103 ± 0.008 (Table 2, Dataset 3). In this study, the genetic distance between Anatidae and Dendrocygnidae is significantly different from genetic distances within the Anatidae.

**Table 1:** The genetic distances and standard error estimates among Anseriformes species of mtDNA COI based on K2P model.

	1	2	3	4
1. Anatidae				
2. Dendrocygnidae	0.158 ± 0.021			
3. Anhimade	0.208 ± 0.031	0.190 ± 0.029		
4. Anseranatidae	0.172 ± 0.026	0.161 ± 0.025	0.173 ± 0.032	

1. Anatidae species—*Anas acuta*, *A*. *bahamensis*, *A*. *gibberifrons*, *A*. *crecca*, *A*. *poecilorhyncha*, *A*. *platyrhynchos*, *A*. *laysanensis*, *A*. *superciliosa*, *A*. *falcata*, *A*. *penelope*, *A*. *strepera*, *A*. *discors*, *A*. *platalea*, *A*. *clypeata*, *A*. *querquedula*, *A*. *formosa*, *Amazonetta brasiliensis*, *Tachyeres pteneres*, *Lophonetta specularoides*, *Aythya ferina*, *A*. *Americana*, *A*. *fuligula*, *A*. *marila*, *A*. *affinis*, *Netta rufina*, *Tadorna tadorna*, *T*. *ferruginea*, *Mergus merganser*, *M*. *serrator*, *M*. *squamatus*, *Mergellus albellus*, *Lophodytes cucullatus*, *Anser anser*, *A*. *brachyrhynchus*, *A*. *cygnoides*, *A*. *indicus*, *A*. *rossii*, *A*. *fabalis*, *A*. *albifrons*, *Branta canadensis*, *B*. *sandvicensis*, *B*. *leucopsis*, *B*. *bernicla*, *Cygnus cygnus*, *C*. *columbianus*, *C*. *olor*, *C*. *atratus*;

2. Dendrocygnidae species—*Dendrocygna javanica*, *D*. *viduata*, *D*. *arcuata*, *D*. *eytoni*, *D*. *bicolor*;

3. Anhimade species—*Anhima cornuta*;

4. Anseranatidae species—*Anseranas semipalmata*.

**Table 2:** The genetic distances and standard error estimates among Anatidae species of mtDNA COI based on K2P model.

	1	2	3	4	5	6	7
1. *Anas*							
2. *Aythya*	0.054 ± 0.008						
3. *Tadorna*	0.048 ± 0.009	0.054 ± 0.010					
4. *Mergus*	0.037 ± 0.006	0.055 ± 0.009	0.048 ± 0.009				
5. *Anser*	0.079 ± 0.011	0.107 ± 0.014	0.101 ± 0.014	0.086 ± 0.012			
6. *Branta*	0.063 ± 0.009	0.093 ± 0.013	0.071 ± 0.011	0.072 ± 0.010	0.043 ± 0.008		
7. *Cygnus*	0.050 ± 0.008	0.074 ± 0.010	0.073 ± 0.012	0.050 ± 0.009	0.061 ± 0.010	0.045 ± 0.008	

1. *Anas* species–*Anas acuta*, *A*. *bahamensis*, *A*. *gibberifrons*, *A*. *crecca*, *A*. *poecilorhyncha*, *A*. *platyrhynchos*, *A*. *laysanensis*, *A*. *superciliosa*, *A*. *falcata*, *A*. *penelope*, *A*. *strepera*, *A*. *discors*, *A*. *platalea*, *A*. *clypeata*, *A*. *querquedula*, *A*. *formosa*, *Amazonetta brasiliensis*, *Tachyeres pteneres* and *Lophonetta specularoides*;

2. *Aythya* species–*Aythya ferina*, *A*. *Americana*, *A*. *fuligula*, *A*. *marila* and *A*. *affinis*;

3. *Tadorna* species–*Tadorna tadorna* and *T*. *ferruginea*;

4. *Mergus* species–*Mergus merganser*, *M*. *serrator*, *M*. *squamatus*, *Mergellus albellus* and *Lophodytes cucullatus*;

5. *Anser* species–*Anser anser*, *A*. *brachyrhynchus*, *A*. *cygnoides*, *A*. *indicus*, *A*. *rossii*, *A*. *fabalis* and *A*. *albifrons*;

6. *Branta* species–*Branta canadensis*, *B*. *sandvicensis*, *B*. *leucopsis* and *B*. *bernicla*;

7. *Cygnus* species–*Cygnus cygnus*, *C*. *columbianus*, *C*. *olor* and *C*. *atratus*.

### Phylogenetic reconstructions

**Fig 1:**
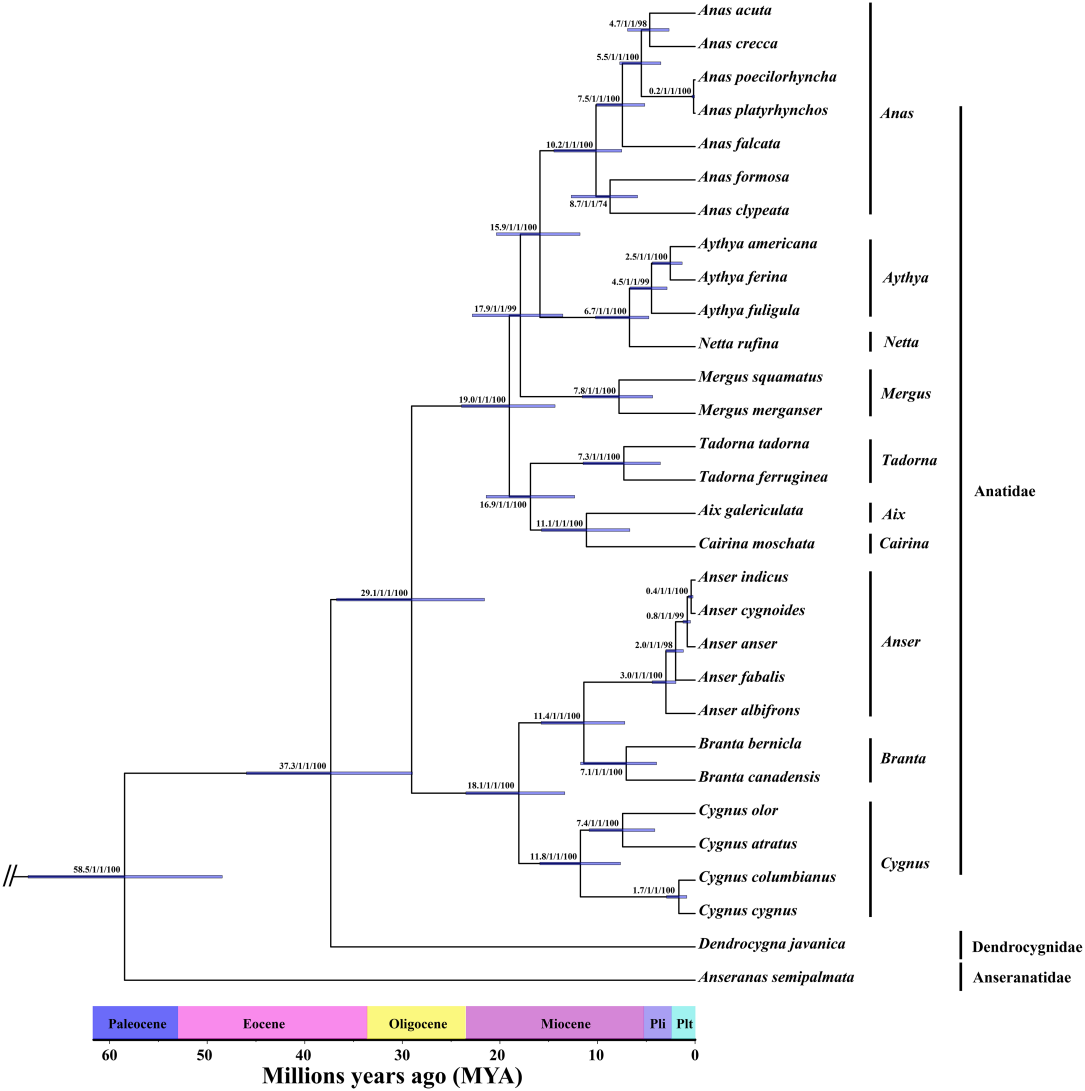
Phylogram showing the phylogenetic relationship in Anseriformes based on the mito-genomes. The values on nodes include four parts. The first two values indicate the split time and Bayesian posterior probabilities which were calculated by BEAST 1.7.4. The third values were the Bayesian posterior probabilities calculated by MrBayes 3.2.2, and the last values were the Bayesian posterior probabilities calculated by RAxML version 8. Blue bars at nodes show 95% highest posterior density (HPD) of divergence times.

**Fig 2:**
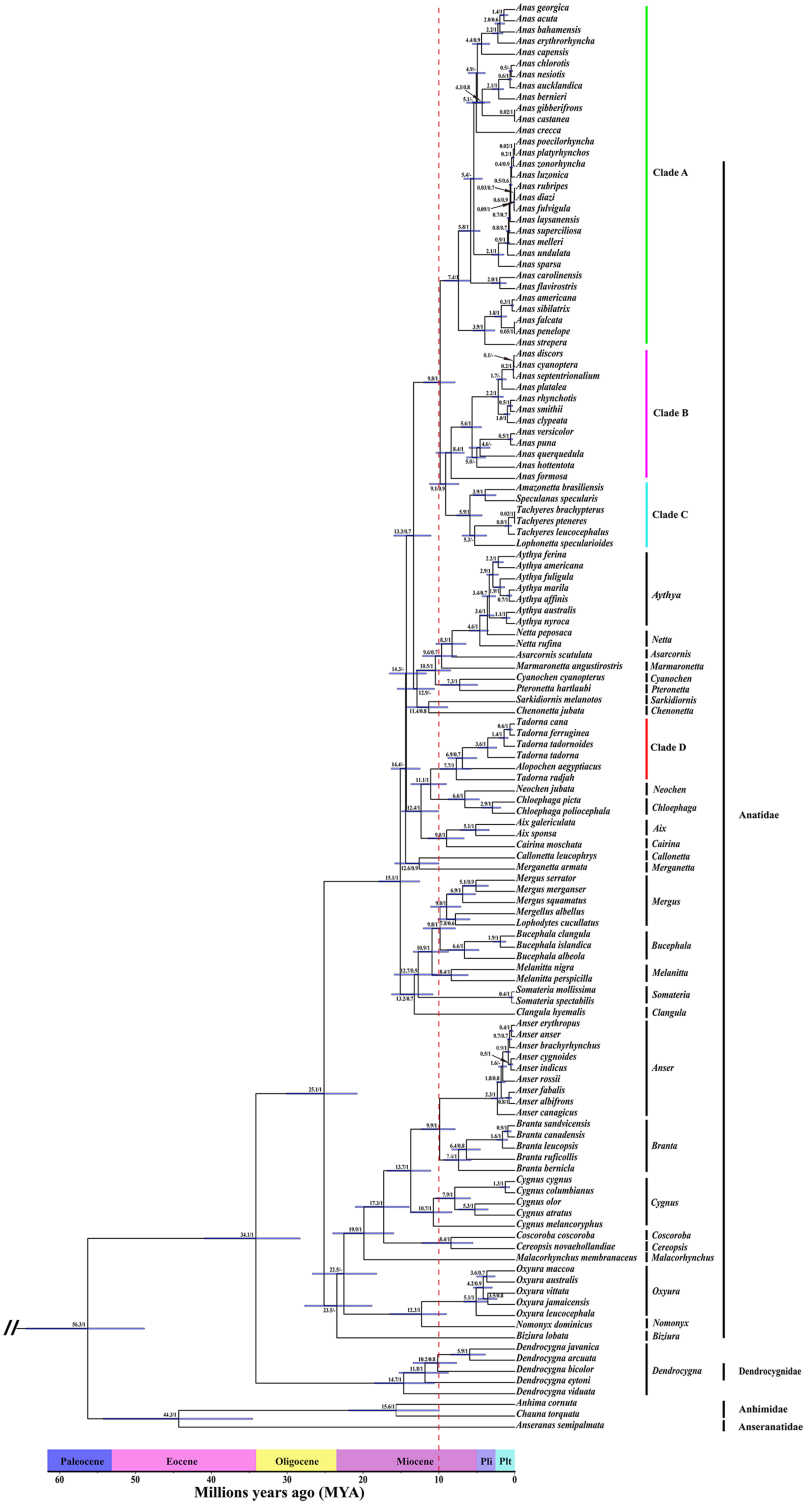
Phylogram showing the phylogenetic relationship in Anseriformes based on two mitochondrial genes. The values on nodes indicate the split time and Bayesian posterior probabilities which were calculated by BEAST 1.7.4, “-” indicated that the value was less than 70. Blue bars at nodes show 95% HPD of divergence times.

The phylogenetic tree recovered from the ML (S2 Fig) and BI (S3 Fig) analyses of mito-genomes for 30 Anseriformes species resulted in the same topology (Fig 1). In this study, the 30 species clustered into two major lineages, represented by Anatidae–Dendrocygnidae and Anseranatidae, respectively (Fig 1). There are three major lineages in the Anatidae–Dendrocygnidae group (Fig 1). Additionally, topologies recovered from the MCMC method of Cyt *b* and ND2 for 128 Anseriformes species obtained highly posterior probability values for most nodes (Fig 2), which shared basic topology with the above results and a previous study [18]. Our study revealed that the 128 Anseriformes species also clustered into two major lineages, represented by Anatidae–Dendrocygnidae and Anhimidae–Anseranatidae, respectively (Fig 2). Furthermore, the Anatidae–Dendrocygnidae group comprises three major lineages (Fig 2).

### Divergence times

In the present study, we obtained similar divergence time values in the major nodes using a molecular clock based on mito-genomes and two concatenated mitochondrial genes. The divergence time between the Anseranatidae–Anhimidae group and Anatidae–Dendrocygnidae group was at about 58.5 Ma (95% HPD: 48.5–68.4, Dataset 1) (Fig 1 and S4 Fig) or 56.3 Ma (95% HPD: 48.8–64.5, Dataset 2) (Fig 2 and S5 Fig). Moreover, the split time between Anseranatidae and Anhimidae was estimated at about 44.3 Ma (95% HPD: 34.6–54.3, Dataset 2) (Fig 2 and S5 Fig). In addition, the Anatidae has split from Dendrocygnidae at about 37.3 Ma (95% HPD: 29.0–46.0, Dataset 1) (Fig 1 and S4 Fig) or 34.1 Ma (95% HPD: 28.3–40.9, Dataset 2) (Fig 2 and S5 Fig).

### Tempo and rate shifts of diversification

**Fig 3:**
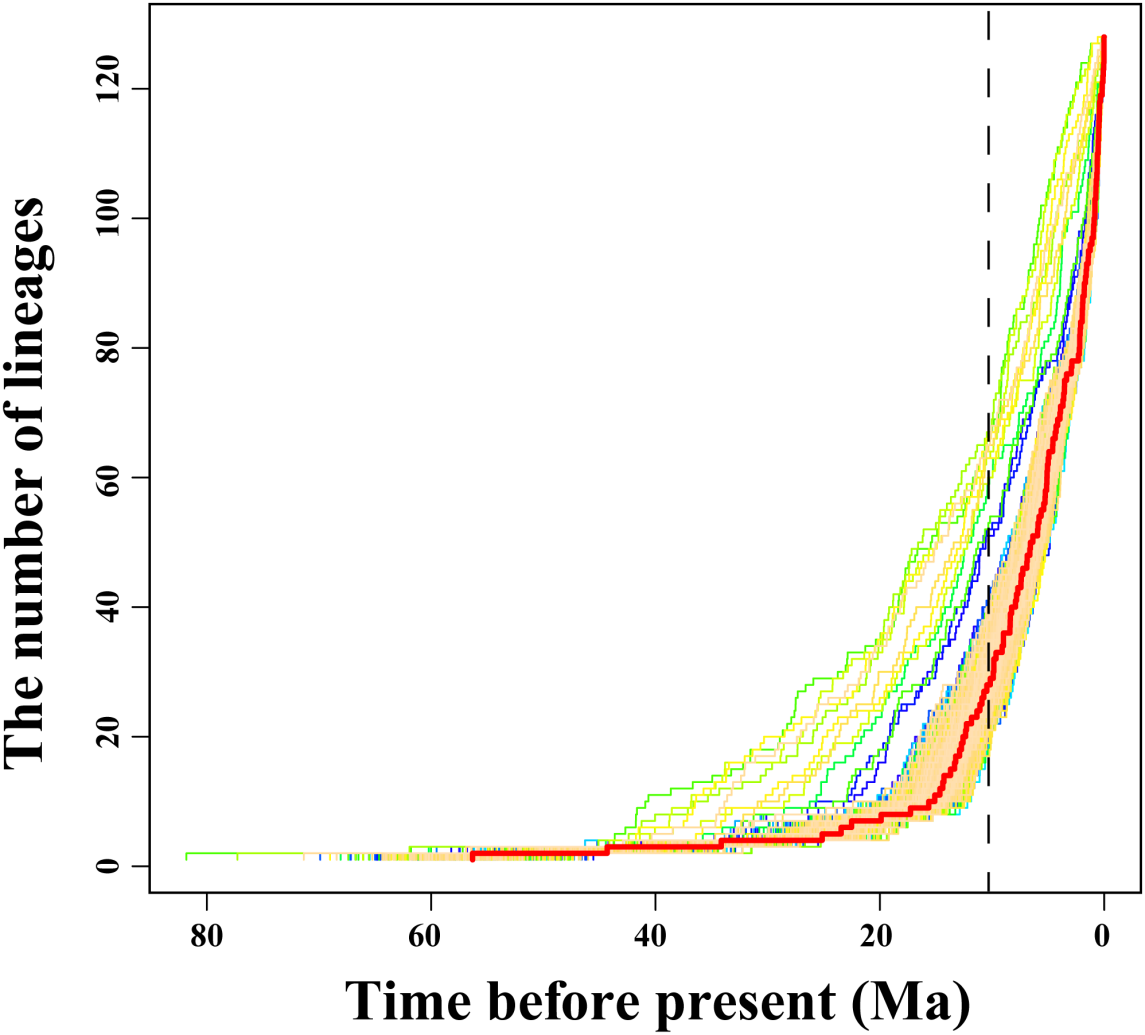
Lineage-through-time (LTT) plots for Anseriformes birds. The colored lines represent the results of 1000 trees randomly selected from the BEAST analysis. The ref line shows the MCC tree.

**Fig 4:**
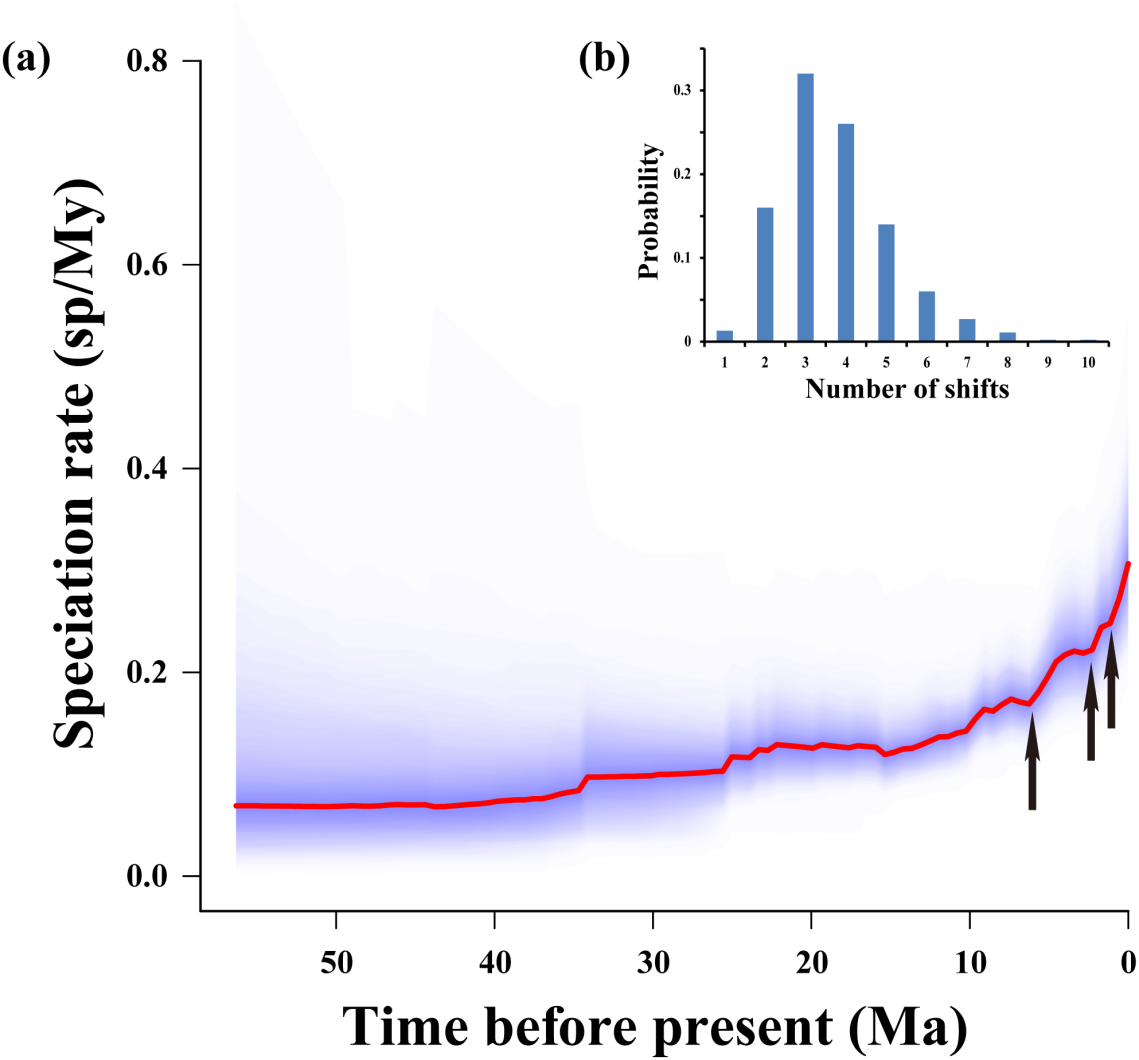
Visualizations of diversification rate shifts within Anseriformes. (a) Speciation-through-time plots utilizing BAMM. Curved red lines represent the median values with the 95% confidence intervals shown in blue. Arrows point to the three significant shifts in rates of speciation; (b) Posterior distribution of the number of rate shifts.

The empirical LTT plot displayed that the diversification rate of the Anseriformes had a significant increase during the late Miocene after a long period of constant diversification (Fig 3). In the BAMM analyses, we confirmed convergence of the MCMC chains after discarding the burn-in, according to the result of effective samples size (ESS is 900). In the Anseiformes, the 95% credible set of rate shift configurations with the highest probability included three or more core shifts (Fig 4a). The best configuration detected three shifts within the Anseriformes birds (Fig 4b), suggesting that the mean initial speciation rate was accelerated three times, and finally increased to approximately 0.3 sp/My in their evolutionary history (Fig 4a).

## Discussion

### Classification implications based on phylogenetic relationships

In the last few decades, the notion of dividing the Anseriformes into three families (Anatidae, Anhimidae and Anseranatidae) was widely prevalent [19, 35–40]. In 1990, Sibley and Ahlquist classified *Dendrocygna* species into an independent family (Dendrocygnidae) based on DNA-DNA hybridization [42]. However, it was not accepted widely [43]. Based on the tree of two concatenated mitochondrial genes, the Anhimidae clustered together with Anseranatidae, which contains a limited number of species (3 species). Meanwhile, the Dendrocygnidae clustered together with Anatidae, which comprises the majority of species (approximately 125 species). In this study, both trees (mito-genomes, Fig 1 and two concatenated mitochondrial genes, Fig 2) showed that Dendrocygnidae is an independent clade, which diverged in the late Eocene at about 37.3 / 34.1 Ma BP (Figs 1 and 2). In birds, the COI gene is considered as standard for DNA barcoding [108]. For the COI gene, the mean K2P distances within-species, genus and family are 0.43%, 7.93% and 12.71%, respectively [108]. In this study, K2P distances between Dendrocygnidae and the other three families (Anatidae, Anhimade and Anseranatidae) of species ranged from 15.8% to 19.0% (Table 1), which are higher than the average genetic distances within the family. In addition, the mean K2P distance between Anatidae and Dendrocygnidae is 15.8% (Table 1), which is significantly different from the mean K2P distance within the Anatidae (10.3%, Table 2). Therefore, our study provide support for Dendrocygnidae to be an independent family, rather than as a genus within Anatidae.

The crested duck (*Lophonetta specularioides*) and spectacled duck (*Speculanas specularis*) were once considered as members of the genus *Anas* [109–111]. In this study, six species from genera *Amazonetta*, *Speculanas*, *Tachyeres* and *Lophonetta* clustered in a distinct clade (Clade C, Fig 2), which is the sister group to Clade B (Fig 2). Meanwhile, Clade B is comprised of twelve *Anas* species (Fig 2). Besides, Clade B and C are sister groups to Clade A, which is comprised of 31 *Anas* species (Fig 2). Based on phylogenetic relationships, the extant *Anas* species (Clade A and B) do not form a monophyletic group. Therefore, in systematics, we suggest that placing these six species (Clade C) into the genus *Anas* would be more appropriate.

The Egyptian goose (*Alopochen aegyptiacus*) is the only extant species in the genus *Alopochen* [110, 111]. Based on the phylogenetic analysis, *Alopochen aegyptiacus* nested tightly together with the *Tadorna* species in Clade D, rather than being an independent clade (Fig 2). It is obvious that *Alopochen aegyptiacus* is a member of the genus *Tadorna*. What’s more, the high incidence of hybridization between *Alopochen aegyptiaca* and *Tadorna tadorna* also suggest the close genetic proximity between *Alopochen* and *Tadorna* [112]. Therefore, we propose that *Alopochen aegyptiacus* should be placed in the genus *Tadorna*.

### Ancestral diversification during the Early Eocene Climatic Optimum

Compared to the members of Anhimidae and Anseranatidae, Anatidae and Dendrocygnidae species have well developed feet webs which helped them become successful swimmers [43]. They tend to exploit different ecological niches, which eventually generates ecological adaptation in their evolutionary history. For example, the Anatidae and Dendrocygnidae species exist in various types of lacustrine systems with a worldwide distribution, except for the South Pole, while the Anhimidae (found in South America) and Anseranatidae (Australia, Indonesia and Papua New Guinea) species have a narrower distribution [30, 32, 33, 35]. Our study identified the ancestral diversification between the Anhimidae–Anseranatidae group and Anatidae–Dendrocygnidae group occurred at about 58.5 / 56.3 Ma (Figs 1 and 2) in the late Paleocene. Furthermore, we compared this estimated divergence times with other studies [113–115] using TimeTree (http://timetree.org/) [116], which also proved that these estimates are reliable.

From the late Paleocene (~ 58 Ma) to the early Eocene (~ 50 Ma), the earth’s surface experienced a long-term warming trend that culminated in an extended period of extreme warmth called the Early Eocene Climatic Optimum (EECO) [117–120]. Remarkably, at the boundary between the Paleocene and Eocene epochs (~ 56 Ma), a most intense and abrupt interval of global warming occurred, called the Paleocene–Eocene Thermal Maximum (PETM) [121–124]. During the EECO interval, the mean annual temperature and the mean annual rainfall increased significantly, while these climate changes promoted a major increase in floral and faunal diversity [118, 125, 126]. For example, they have induced diversification in many animal groups, such as insects [127], salamanders [128], bats [10] and the ruminants [12]. In this study, this ancestral diversification in Anseriformes occurred close to the PETM during the EECO. Therefore, we suggest that the warmer climatic conditions during the EECO induced this ancestral diversification in Anseriformes, which reinforced their habit of exploiting different ecological niches.

### Rapid and recent diversification during the Plio-Pleistocene

Diversification patterns and other potential driving factors, together with accurate divergence time estimation can be used to predict species change [129]. Therefore, we performed a test to characterize the patterns of diversification of this group by using the molecular phylogeny as a robust framework. Based on the results of divergence time estimation and LTT plot analyses, rapid and recent diversification events can be observed to have occurred in the Anseriformes since 10.0 Ma in the Late Miocene (Figs 2 and 3). Clearly, a higher speciation rate has characterized the rapid and recent diversification in the Anseriformes during the Plio-Pleistocene, while the mean initial speciation rate accelerated three times, and finally increased to approximately 0.3 sp/My (Fig 4). Specifically, the Anatidae comprises the majority of species (approximately 148), which diversified at about 29.1 / 25.1 Ma (Figs 1 and 2). Therefore, we proposed that the Anatidae species might have experienced rapid and recent diversification during the Plio-Pleistocene.

In terrestrial and lacustrine systems, mechanisms of speciation are thought to be important for ecological adaptation [8]. Many speciation events showed that some underlying factors, such as the availability of new resources [2], new habitats [130], or key innovations that promote the exploitation of new resources or habitats, promoted such rapid radiation [131]. In this study, three rapid and recent diversification events have been detected during the Plio-Pleistocene (Fig 4). Among of them, the first acceleration event occurred during the late Pliocene, while the second and third acceleration event occurred during the Pleistocene [132]. During the Plio-Pleistocene intervals, the climate fluctuations induced geographic isolation, habitat fragmentation and changes in resource availability, which acted in concert to produce great species diversity and richness [125, 133–138]. Therefore, Plio-Pleistocene climate fluctuation fostered rapid and recent diversification in many taxonomic groups, such as the invertebrates (e.g. *Sternopriscus* species, [134]; *Kikihia* species, [139]; *Warramaba* species, [140]; *Pseudovelia* species, [141]); amphibians (e.g. *Scinax* species, [142]; *Hypsiboas* species, [143]); reptiles (e.g. *Eulamprus* species, [144]; *Drysdalia* species, [145]; *Montivipera* species, [146]) and mammals (e.g. *Myosorex* species, [147]; *Nannomys*, *Aethomys*, *Otomys*, *Myotomys*, *Rhabdomys*, *Mastomys*, *Saccostomus*, *Cryptomys* and *Xerus* species, [148]; *Mustela* species, [149]). Notably, whether the Pleistocene events caused substantial avian diversification has been a matter of long-standing debate [150]. However, many studies proved that the Pleistocene climate cycling played a primary role in driving speciation within many avian species [135, 151–155]. Particularly, many waterfowls (e.g., *Mesitornis unicolor*, *Nipponia Nippon*, *Pelecanus crispus* and *Balearica regulorum*) have undergone rapid and recent diversification during the Pleistocene [155]. Besides, the Plio-Pleistocene climate fluctuation have also driven the rapid speciation events in *Anser* species [25]. Therefore, we proposed that the Plio-Pleistocene climate fluctuation have also played a significant role in diversification of Anseriformes birds.

## Conclusion

In this study, we sequenced and annotated nine mito-genomes of Anseriformes birds, which ranged from 16,599 bp to 16,630 bp. In addition we have included two mitochondrial genes and reconstructed a strongly supported phylogeny, which covered the majority species (128 species, 39 genera) in Anseriformes. Based on the results of molecular phylogeny and genetic distances, we revised the classification of the *Dendrocygna*, *Amazonetta*, *Speculanas*, *Tachyeres*, *Lophonetta* and *Alopochen*. Furthermore, we strongly suggested that the *Dendrocygna* species should be considered as an independent family (Dendrocygnidae). During the EECO (from 58 to 50 Ma), the warmer climatic conditions induced the ancestral diversification in Anseriformes, which reinforced their habit of exploiting different ecological niches. At last, our study provided evidence to support that the Plio-Pleistocene climate fluctuation fostered the rapid and recent diversification in Anseriformes, especially in Anatidae.

## Supporting information

S1 Table Primers designed in this study. (DOCX)

S2 Table GenBank accession numbers for the 30 complete mtDNA of Anseriformes species in this study. (DOCX)

S3 Table GenBank accession numbers for the 128 ND2 and Cyt *b* of Anseriformes species in this study. (DOCX)

S4 Table GenBank accession numbers for the 54 COI gene of Anseriformes species in this study. (DOCX)

S5 Table Five calibration points used in the divergence time analyses. (DOCX)

S6 Table Nucleotide composition (%) of some Anseriformes mitochondrial genomes. (DOCX)

S1 Fig Graphical map of complete mitochondrial genome of Anatidae species. Genes encoded by the heavy strand were shown outside the circle, and encoded by the light strand were shown inside the circle respectively. The inner ring showed the general GC content of the complete mitochondrial genome sequence.
(TIF)

S2 Fig RAxML tree in Anseriforme based on complete mtDNA. The nodal numbers are posterior probabilities.
(TIF)

S3 Fig Bayesian inference tree in Anseriforme based on complete mtDNA. The nodal numbers are posterior probabilities.
(TIF)

S4 Fig Divergence times for the major clades of Anseriformes based on complete mtDNA and five calibration points (A-E) for dating analyses. (TIF)

S5 Fig Divergence times for the major clades of Anseriformes based on two mitochondrial genes and five calibration points (A-E) for dating analyses. (TIF)
