# Supplementary material for: Rapid and recent diversification patterns in Anseriformes birds: Inferred from molecular phylogeny and diversification analyses
Source: PLoS One. 2017 Sep 11;12(9):e0184529. doi: 10.1371/journal.pone.0184529 (PMC5593203; doi:10.1371/journal.pone.0184529)
Supplement: S1 Table — (DOCX) [file pone.0184529.s001.docx]

**S1Table. Sixteen pairs of primers designed in this study.**

| Primer | Forward primer sequence (5’→3’) | Reverse primer sequence (5’→3’) | Amplified region | Product size (bp) | Annealing  temperature (°C) |
| --- | --- | --- | --- | --- | --- |
| Ya-1 | AGCATGGCACTGAAGCTGC | CTTTTCTATCGCCTATACTAGGAC | 40-1224 | 1184 | 55 |
| Ya-2 | GGTAAGTGTACCGGAAGGTG | CCACAGGGTCTTCTCGTC | 1055-2217 | 1160 | 50 |
| Ya-3 | AATCTGTGAAAGGAACTCGGC | ATCATAGGATTGAGTAGACGGC | 1971-3196 | 1225 | 52 |
| Ya-4 | TCATTAAAGAGCCCATTCGACC | TTGCGGCTTCTACTGCTCG | 3050-4187 | 1137 | 53 |
| Ya-5 | CCATACCCCGGAAATGATGG | CGAAGCCCGTCTGTCTAG | 4016-5210 | 1194 | 53 |
| Ya-6 | CACGCCCACCGCAATCCT | GATGGATAGCATGGCTCAGAC | 5002-6244 | 1242 | 54 |
| Ya-7 | GATTCTTCGGCCACCCAG | GGCTTTTAGTGTGAGGTCTGG | 6148-7382 | 1234 | 53 |
| Ya-8 | GAATTTCACGACCACGCTCTG | GGCTAAGGAGGCTGATGG | 7129-8389 | 1260 | 53 |
| Ya-9 | CGGTGAATCAACAACCGACT | TTAGTCGGAGGAGGCAGAC | 8152-9358 | 1206 | 52 |
| Ya-10 | ACATGAGCCCACCACAGC | CATTGGAGGCTGAGGGCG | 9159-10380 | 1221 | 53 |
| Ya-11 | CTAGCCATCCTAGTAGCCTC | ATGCGGTTAGTAGGGTGGC | 10227-11470 | 1243 | 53 |
| Ya-12 | CCTCCTCCATACTATTCTGCC | GTGGGGGTGTACGGCTTG | 11260-12462 | 1202 | 54 |
| Ya-13 | CCTACTAACATTCCTAATTGCCATG | CGATTAGGTGTAGGGCGATG | 12248-13502 | 1254 | 54 |
| Ya-14 | TAATCACCTCCTTCATCACACC | TAATGTGTGGTGGGGTTACTAG | 13282-14468 | 1186 | 53 |
| Ya-15 | CCCAACCCTAACCCGATTC | GGCCGAGCAGGAATAATGAC | 14218-15503 | 1285 | 53 |
| Ya-16 | CACAAGCGCCGCCGCAT | ATTTACACTGGGGCGCGG | 15239-127 | 1537 | 53 |
