# Supplementary material for: Rapid and recent diversification patterns in Anseriformes birds: Inferred from molecular phylogeny and diversification analyses
Source: PLoS One. 2017 Sep 11;12(9):e0184529. doi: 10.1371/journal.pone.0184529 (PMC5593203; doi:10.1371/journal.pone.0184529)
Supplement: S2 Table — (DOCX) [file pone.0184529.s002.docx]

**S1 Table. GenBank accession numbers for the 30 complete mtDNA of Anseriformes species in this study.**

| Species | Accession number | Species | Accession number |
| --- | --- | --- | --- |
| *Anas acuta* | KF312717 (in this study) | *Cairina moschata* | NC_010965 |
| *Anas crecca* | KF203133 (in this study) | *Aix galericulata* (in this study) | KJ169568 |
| *Anas poecilorhyncha* | KF156760 (in this study) | *Anser indicus* | NC_025654 |
| *Anas platyrhynchos* | EU009397 | *Anser cygnoides* | NC_023832 |
| *Anas falcata* | NC_023352 | *Anser anser* | NC_011196 |
| *Anas formosa* | NC_015482 | *Anser fabalis* | NC_016922 |
| *Anas clypeata* | KT345702 (in this study) | *Anser albifrons* | NC_004539 |
| *Aythya americana* | NC_000877 | *Branta bernicla* | KJ680301 |
| *Aythya ferina* | KJ710708 (in this study) | *Branta canadensis* | NC_007011 |
| *Aythya fuligula* | KJ722069 (in this study) | *Cygnus olor* | NC_027096 |
| *Netta rufina* | NC_024922 | *Cygnus atratus* | NC_012843 |
| *Mergus squamatus* | NC_016723 | *Cygnus columbianus* | NC_007691 |
| *Mergus merganser* | KU140667 (in this study) | *Cygnus cygnus* | NC_027095 |
| *Tadorna tadorna* | KU140668 (in this study) | *Dendrocygna javanica* | NC_012844 |
| *Tadorna ferruginea* | NC_024640 | *Anseranas semipalmata* | NC_005933 |
