# Supplementary material for: Rapid and recent diversification patterns in Anseriformes birds: Inferred from molecular phylogeny and diversification analyses
Source: PLoS One. 2017 Sep 11;12(9):e0184529. doi: 10.1371/journal.pone.0184529 (PMC5593203; doi:10.1371/journal.pone.0184529)
Supplement: S3 Table — (DOCX) [file pone.0184529.s003.docx]

**S2 Table. GenBank accession numbers for the 128 Anseriformes species in this study.**

| Species | Accession number (Cyt b, ND2) | Species | Accession number (Cyt b, ND2) |
| --- | --- | --- | --- |
| *Mergus squamatus* | NC_016723, NC_016723 | *Aythya fuligula* | KJ722069, KJ722069 |
| *Mergus merganser* | KU140668, KU140668 | *Aythya affinis* | EU585621, EU585684 |
| *Mergus serrator* | EU585655, EU585718 | *Aythya marila* | EU585625, EU585688 |
| *Mergellus albellus* | EU585653, EU585716 | *Aythya australis* | EU585622, EU585685 |
| *Lophodytes cucullatus* | EU585650, EU585713 | *Aythya nyroca* | EU585626, EU585689 |
| *Bucephala albeola* | EU585633, EU585696 | *Netta peposaca* | EU585656, EU585719 |
| *Bucephala clangula* | EU585634, EU585697 | *Netta rufina* | NC_024922, NC_024922 |
| *Bucephala islandica* | EU585635, EU585698 | *Asarcornis scutulata* | AF059099, AF059159 |
| *Melanitta nigra* | AF515263, AF515267 | *Marmaronetta angustirostris* | AF059104, AF059164 |
| *Melanitta perspicilla* | EU585652, EU585715 | *Pteronetta hartlaubi* | AF059110, AF059170 |
| *Somateria spectabilis* | EU585662, EU585725 | *Cyanochen cyanopterus* | EU585640, EU585703 |
| *Somateria mollissima* | AF515264, AF515268 | *Chenonetta jubata* | AF059100, AF059160 |
| *Clangula hyemalis* | EU585638, EU585701 | *Sarkidiornis melanotos* | EU585660, EU585723 |
| *Speculanas specularis* | HM063572, AF059150 | *Callonetta leucophrys* | AF059097, AF059157 |
| *Amazonetta brasiliensis* | HM063571, AF059115 | *Merganetta armata* | HM063579, HM063566 |
| *Tachyeres leucocephalus* | HM063560, HM063569 | *Tadorna ferruginea* | NC_024640, NC_024640 |
| *Tachyeres pteneres* | AF059112, AF059172 | *Tadorna cana* | EU585663, EU585726 |
| *Tachyeres brachypterus* | HM063574, HM063559 | *Tadorna tadornoides* | EU585666, EU585729 |
| *Lophonetta specularioides* | AF059102, AF059162 | *Tadorna tadorna* | KU140668, KU140668 |
| *Anas versicolor* | AF059094, AF059154 | *Alopochen aegyptiacus* | HM063576, HM063563 |
| *Anas puna* | EU585611, EU585674 | *Tadorna radjah* | EU585665, EU585728 |
| *Anas querquedula* | EU585610, AF059146 | *Neochen jubata* | HM063577, HM063564 |
| *Anas hottentota* | EU585608, EU585671 | *Chloephaga poliocepha* | EU585637, EU585700 |
| *Anas septentrionalium* | AF059067, AF059127 | *Chloephaga picta* | AF515262, AF515266 |
| *Anas cyanoptera* | EU914155, AF059127 | *Aix galericulata* | KJ169568, KJ169568 |
| *Anas discors* | AF059068, AF059128 | *Aix sponsa* | AF059053, AF059114 |
| *Anas platalea* | AF059084, AF059144 | *Cairina moschata* | NC_010965, NC_010965 |
| *Anas rhynchotis* | AF059087, AF059147 | *Anser indicus* | NC_025654, NC_025654 |
| *Anas smithii* | AF059089, AF059149 | *Anser cygnoides* | NC_023832, NC_023832 |
| *Anas clypeata* | KT345702, KT345702 | *Anser brachyrhynchus* | EU585614, EU585677 |
| *Anas formosa* | NC_015482, NC_015482 | *Anser anser* | NC_011196, NC_011196 |
| *Anas georgica* | AF059075, AF059135 | *Anser erythropus* | EU161872, EU585680 |
| *Anas acuta* | KF312717, KF312717 | *Anser rossii* | EU585620, EU585683 |
| *Anas bahamensis* | AF059059, AF059120 | *Anser albifrons* | NC_004539, NC_004539 |
| *Anas erythrorhyncha* | AF059070, AF059130 | *Anser fabalis* | NC_016922, NC_016922 |
| *Anas capensis* | AF059105, AF059165 | *Anser canagica* | EU585615, EU585678 |
| *Anas castanea* | AF059065, AF059125 | *Branta ruficollis* | EU585631, EU585694 |
| *Anas gibberifrons* | AF059076, AF059136 | *Branta bernicla* | NC_027066, NC_027066 |
| *Anas bernieri* | AF059060, AF059121 | *Branta leucopsis* | EU585630, EU585693 |
| *Anas nesiotis* | AF059118, AF059057 | *Branta sandvicensis* | EU585632, EU585695 |
| *Anas aucklandica* | AF059056, AF059117 | *Branta canadensis* | NC_007011, NC_007011 |
| *Anas chlorotis* | AF059061, AF059122 | *Cygnus melancoryphus* | EU585644, EU585707 |
| *Anas diazi* | AF059069, AF059129 | *Cygnus cygnus* | NC_027095, NC_027095 |
| *Anas rubripes* | AF059088, AF059148 | *Cygnus columbianus* | NC_007691, NC_007691 |
| *Anas fulvigula* | AF059074, AF059134 | *Cygnus olor* | NC_027096, NC_027096 |
| *Anas poecilorhyncha* | KF156760, KF156760 | *Cygnus atratus* | NC_012843, NC_012843 |
| *Anas platyrhynchos* | EU009397, EU009397 | *Cereopsis novaehollandiae* | EU585636, EU585699 |
| *Anas zonorhyncha* | AF059095, AF059155 | *Coscoroba coscoroba* | EU585639, EU585702 |
| *Anas luzonica* | AF059079, AF059139 | *Malacorhynchus membranaceus* | EU585651, EU585714 |
| *Anas laysanensis* | AF059078, AF059138 | *Oxyura jamaicensis* | EU585658, EU585721 |
| *Anas superciliosa* | AF059092, AF059152 | *Oxyura vittata* | AY747859, AY747865 |
| *Anas melleri* | AF059080, AF059140 | *Oxyura maccoa* | AY747863, AY747869 |
| *Anas undulata* | AF059093, AF059153 | *Oxyura australis* | AY747861, AY747867 |
| *Anas sparsa* | AF059091, AF059151 | *Oxyura leucocephala* | AY747862, AY747868 |
| *Anas flavirostris* | AF059072, AF059132 | *Nomonyx dominicus* | AF119165, AY747864 |
| *Anas carolinensis* | AF059063, AF059123 | *Biziura lobata* | EU585627, EU585690 |
| *Anas falcata* | NC_023352, NC_023352 | *Dendrocygna javanica* | NC_012844, NC_012844 |
| *Anas penelope* | EU914158, HM640875 | *Dendrocygna arcuata* | AF082061, U97735 |
| *Anas americana* | AF059103, HM640867 | *Dendrocygna bicolor* | EU585646, EU585709 |
| *Anas sibilatrix* | AF059108, AF059168 | *Dendrocygna eytoni* | EU585647, EU585710 |
| *Anas strepera* | EU574791, AF059169 | *Dendrocygna viduata* | EU585649, EU585712 |
| *Anas crecca* | KF203133, KF203133 | *Anseranas semipalmata* | NC_005933, NC_005933 |
| *Aythya ferina* | KJ710708, KJ710708 | *Chauna torquata* | AY274030, AY274053 |
| *Aythya americana* | NC_000877, NC_000877 | *Anhima cornuta* | AY140735, AY140737 |
