# Supplementary material for: Rapid and recent diversification patterns in Anseriformes birds: Inferred from molecular phylogeny and diversification analyses
Source: PLoS One. 2017 Sep 11;12(9):e0184529. doi: 10.1371/journal.pone.0184529 (PMC5593203; doi:10.1371/journal.pone.0184529)
Supplement: S4 Table — (DOCX) [file pone.0184529.s004.docx]

**S4 Table. GenBank accession numbers for the 54 COI gene of Anseriformes species in this study.**

| Species | Accession number | Species | Accession number |
| --- | --- | --- | --- |
| *Anas acuta* | NC_024631 | *Mergus merganser*, | KU140667 |
| *A. bahamensis* | FJ027081 | *M. serrator* | AY666208 |
| *A. gibberifrons* | JQ174015 | *M. squamatus* | NC_016723 |
| *A. crecca* | KF203133 | *Mergellus albellus* | GU571480 |
| *A. poecilorhyncha* | KF156760 | *Lophodytes cucullatus* | DQ434635 |
| *A. platyrhynchos* | EU009397 | *Anser anser* | NC_011196 |
| *A. laysanensis* | JF498830 | *A. brachyrhynchus* | GU179004 |
| *A. superciliosa* | JN801396 | *A. cygnoides* | NC_023832 |
| *A. falcata* | NC_023352 | *A. indicus* | GU179002 |
| *A. penelope* | JN703207 | *A. rossii* | DQ434538 |
| *A. strepera* | GQ481327 | *A. fabalis* | NC_016922 |
| *A. discors* | AY666325 | *A. albifrons* | NC_004539 |
| *A. platalea* | FJ027098 | *Branta canadensis* | NC_007011 |
| *A. clypeata* | KT345702 | *B. sandvicensis* | JF498832 |
| *A. querquedula* | GQ481326 | *B. leucopsis* | GU179003 |
| *A. formosa* | NC_015482 | *B. bernicla* | DQ434345 |
| *Amazonetta brasiliensis* | FJ027065 | *Cygnus cygnus* | NC_027095 |
| *Tachyeres pteneres* | JN802007 | *C. columbianus* | NC_007691 |
| *Lophonetta specularoides* | JN801488 | *C. olor* | NC_027096 |
| *Aythya ferina* | KJ710708 | *C. atratus* | NC_012843 |
| *A. americana* | NC_000877 | *Dendrocygna javanica*, | NC_012844 |
| *A. fuligula* | NC_024595 | *D. viduata* | FJ027500 |
| *A. marila* | DQ434333 | *D. arcuata* | U97735 |
| *A. affinis* | DQ434308 | *D. eytoni* | U97733 |
| *Netta rufina* | NC_024922 | *D. bicolor* | U97732 |
| *Tadorna tadorna* | KU140668 | *Anhima cornuta* | AY140729 |
| *T. ferruginea* | NC_024640 | *Anseranas semipalmata* | NC_005933 |
