# Supplementary material for: Rapid and recent diversification patterns in Anseriformes birds: Inferred from molecular phylogeny and diversification analyses
Source: PLoS One. 2017 Sep 11;12(9):e0184529. doi: 10.1371/journal.pone.0184529 (PMC5593203; doi:10.1371/journal.pone.0184529)
Supplement: S5 Table — (DOCX) [file pone.0184529.s005.docx]

**S5 Table. Five calibration points used in the divergence time analyses.**

| Calibration points | Outgroups | Mean age (Myr) | Height_95%_HPD (Myr) |
| --- | --- | --- | --- |
| A | Neognathae VS Palaeognathae | 101.6 | 97.6-107.8 |
| B | Tinamou VS Ostrich | 83.8 | 58.2-95.2 |
| C | Galliformes VS Anseriformes | 65.8 | 54.0-74.1 |
| D | Galloanseres VS Neoaves | 88.6 | 83.6-94.0 |
| E | Columbea VS Passerea | 69.2 | 65.8-72.4 |
