# Supplementary material for: Rapid and recent diversification patterns in Anseriformes birds: Inferred from molecular phylogeny and diversification analyses
Source: PLoS One. 2017 Sep 11;12(9):e0184529. doi: 10.1371/journal.pone.0184529 (PMC5593203; doi:10.1371/journal.pone.0184529)
Supplement: S6 Table — (DOCX) [file pone.0184529.s006.docx]

**S7 Table. Nucleotide composition (%) of some Anseriformes mitochondrial genomes.**

| Species | A (%) | G (%) | C (%) | T (%) | A+T (%) | G+C (%) | Total nucleotide (bp) |
| --- | --- | --- | --- | --- | --- | --- | --- |
| *Anas acuta* | 28.8 | 16.2 | 32.9 | 22.1 | 50.9 | 49.1 | 16,599 |
| *Anas poecilorhyncha* | 29.1 | 15.8 | 32.8 | 22.2 | 51.3 | 48.7 | 16,608 |
| *Anas crecca* | 29.1 | 16.0 | 32.6 | 22.3 | 51.4 | 48.6 | 16,601 |
| *Anas clypeata* | 29.4 | 15.7 | 32.5 | 22.4 | 51.8 | 48.2 | 16,599 |
| *Aythya ferina* | 29.4 | 15.6 | 32.8 | 22.2 | 51.6 | 48.4 | 16,616 |
| *Aythya fuligula* | 29.4 | 15.5 | 32.9 | 22.2 | 51.6 | 48.4 | 16,616 |
| *Mergus merganser* | 28.7 | 16.2 | 33.2 | 21.9 | 50.6 | 49.4 | 16,630 |
| *Tadorna tadorna* | 29.4 | 15.7 | 33.2 | 21.7 | 51.1 | 48.9 | 16,622 |
| *Aix galericulata* | 29.2 | 15.7 | 32.8 | 22.4 | 51.6 | 48.4 | 16,605 |
