# Supplementary figures and images for: Rapid and recent diversification patterns in Anseriformes birds: Inferred from molecular phylogeny and diversification analyses
Source: PLoS One. 2017 Sep 11;12(9):e0184529. doi: 10.1371/journal.pone.0184529 (PMC5593203; doi:10.1371/journal.pone.0184529)

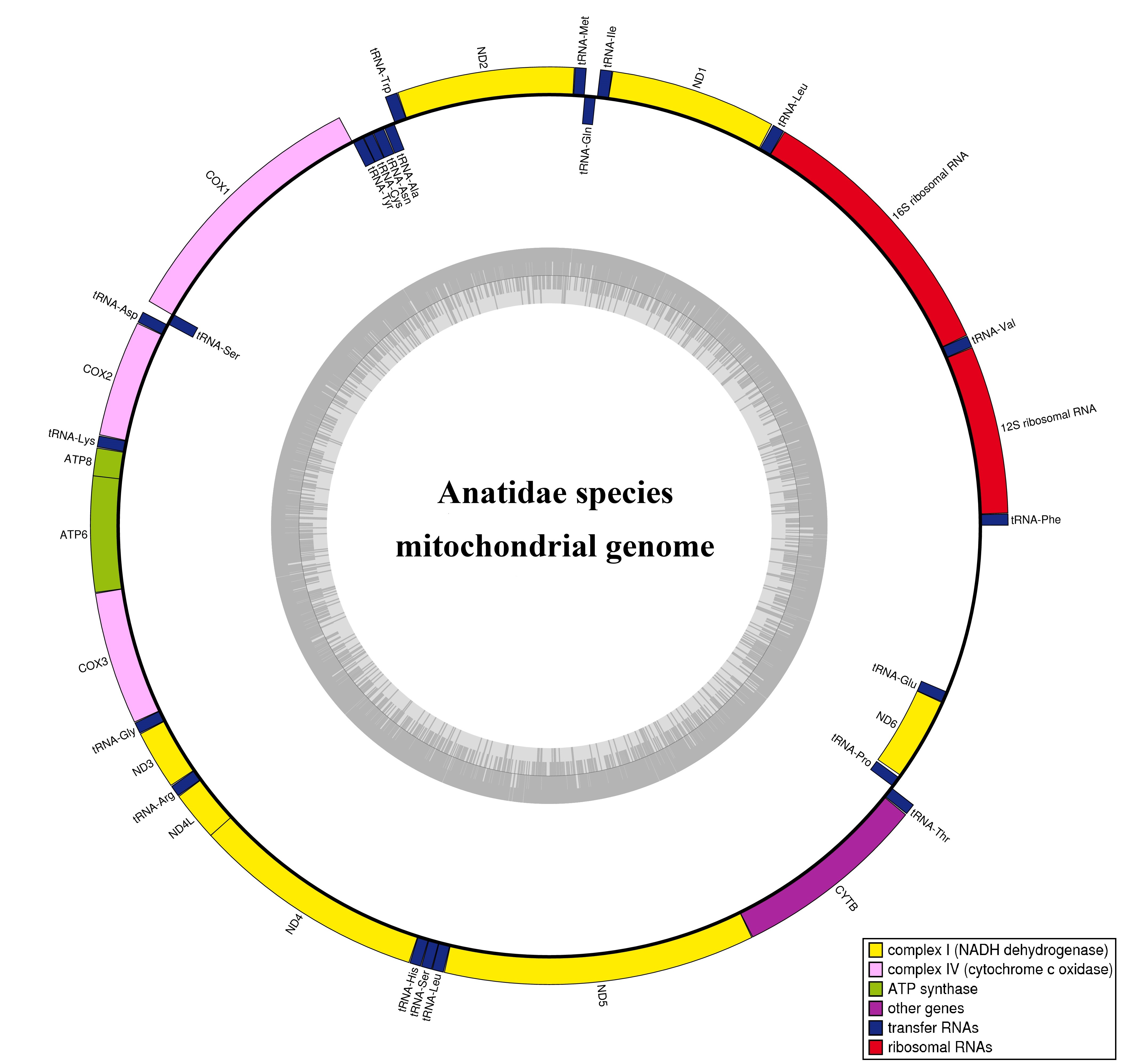

Supplement: S1 Fig — Genes encoded by the heavy strand were shown outside the circle, and encoded by the light strand were shown inside the circle respectively. The inner ring showed the general GC content of the complete mitochondrial genome sequence. (TIF) [file pone.0184529.s007.tif]

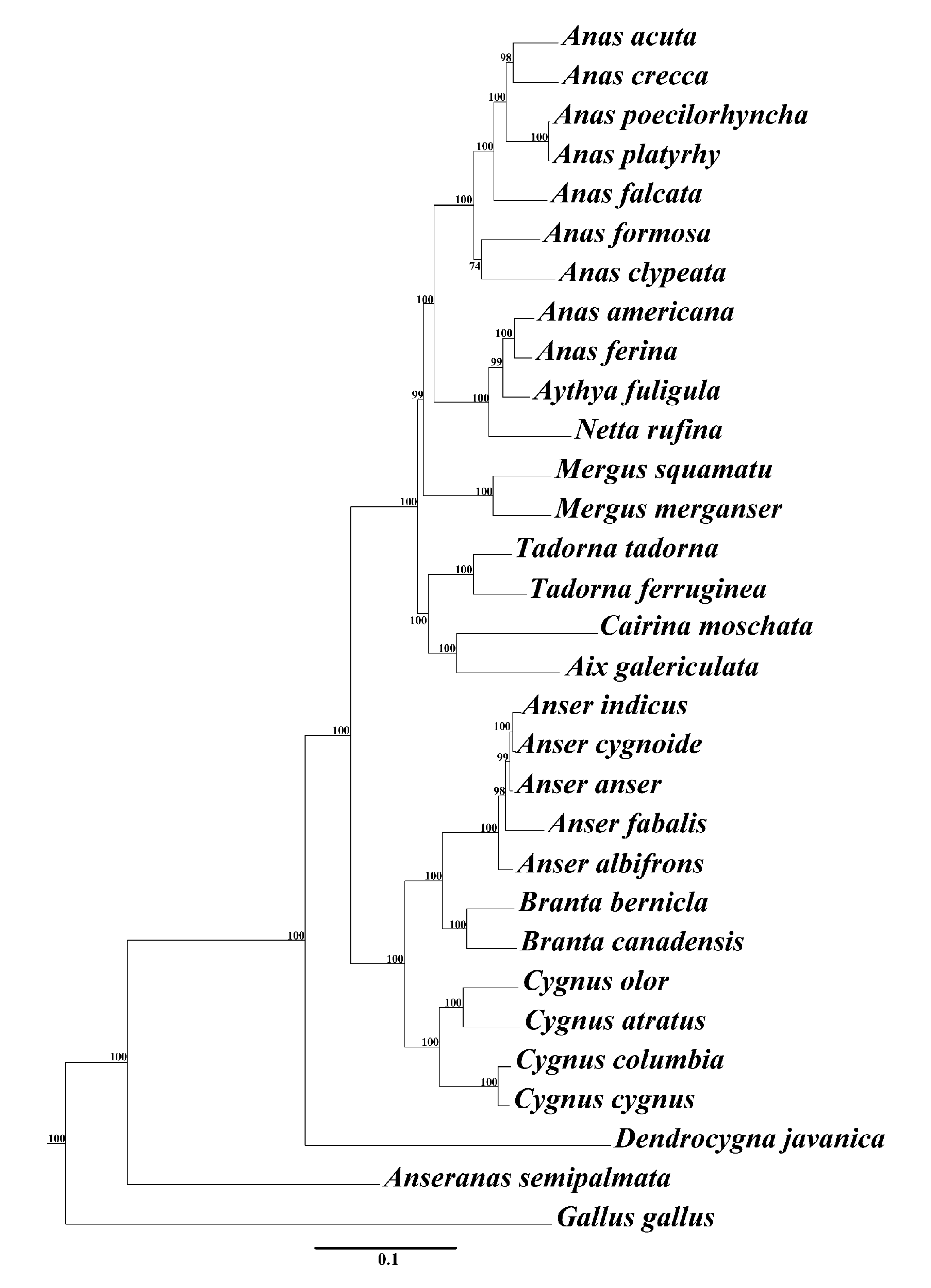

Supplement: S2 Fig — The nodal numbers are posterior probabilities. (TIF) [file pone.0184529.s008.tif]

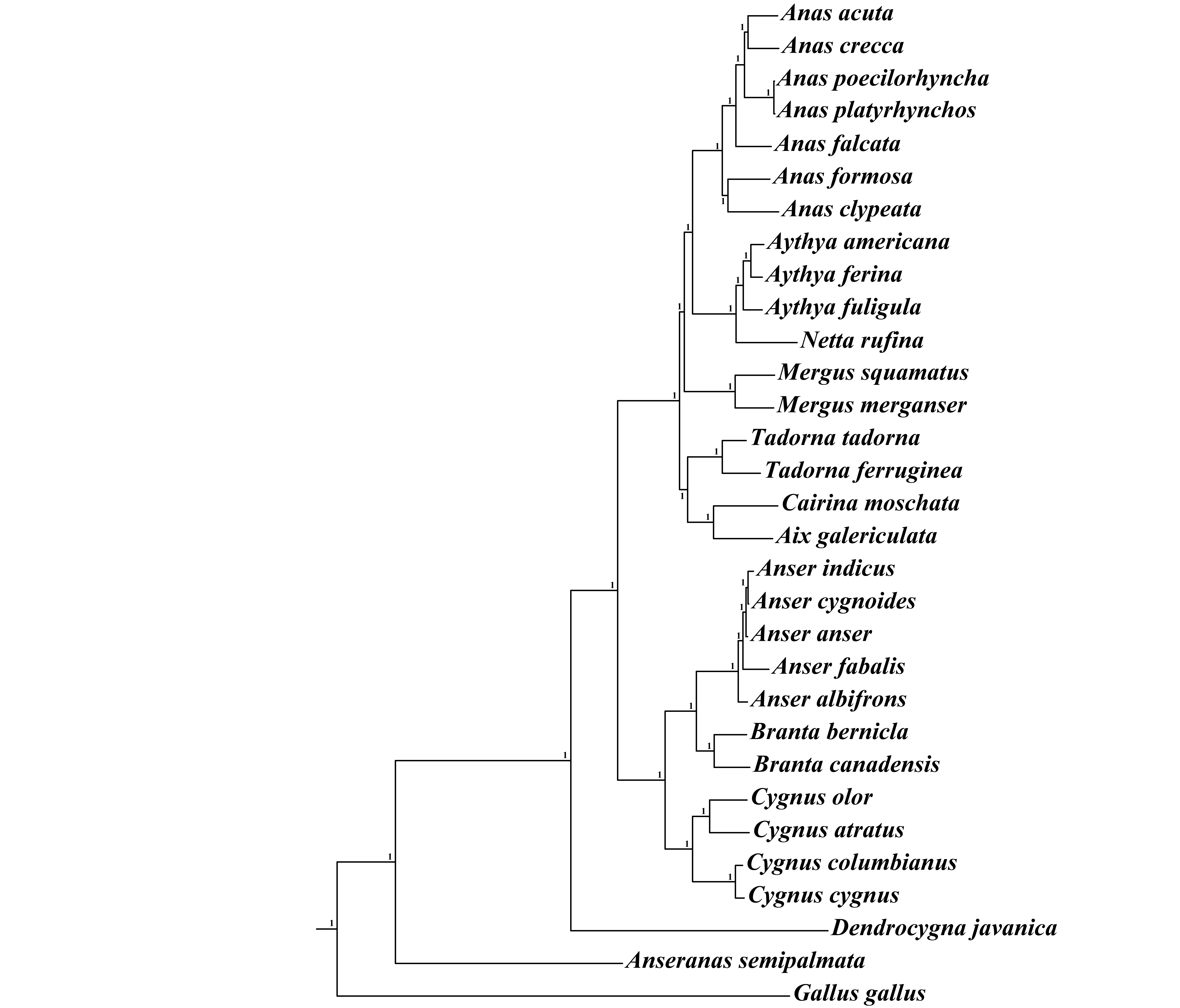

Supplement: S3 Fig — The nodal numbers are posterior probabilities. (TIF) [file pone.0184529.s009.tif]

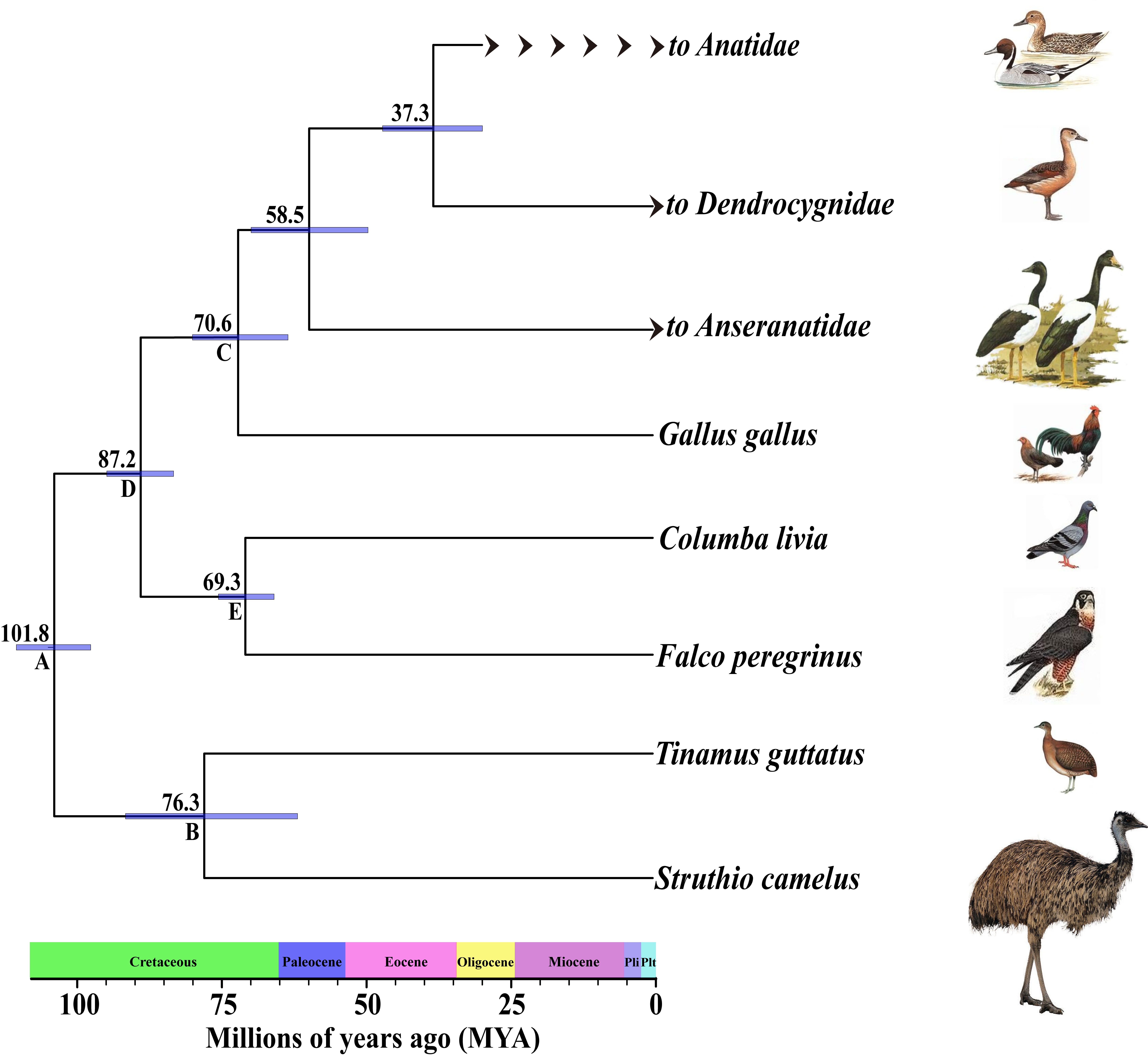

Supplement: S4 Fig — (TIF) [file pone.0184529.s010.tif]
